# Evidence for the existence of pathogenicity determinants in the Long Terminal Repeats (LTRs) of the Bovine Leukemia Virus (BLV) genome

**DOI:** 10.1186/1742-4690-8-S1-A26

**Published:** 2011-06-06

**Authors:** Sabrina M Rodríguez, Karina Trono, Leandro R Jones

**Affiliations:** 1 Molecular and Cellular Epigenetics, Interdisciplinary Cluster for Applied Genoproteomics (GIGA), University of Liège (ULg), Liège, 4000,Belgium; 2 Instituto de Virología, CICVyA, Instituto Nacional de Tecnología Agropecuaria INTA-Castelar, CC 25 (1712), Castelar, Argentina; 3 División de Biología Molecular, Estación de Fotobiología Playa Unión, CC 15, Rawson, Chubut, 9103, Argentina

## 

The majority of BLV-infected animals are asymptomatic carriers (AL) while about 30% develop a benign persistent lymphocytosis (PL). Fatal lymphosarcoma (LS) occurs in 5% of infected animals. The genetic basis of these diverse outcomes of BLV infection is still unknown.

Viral LTRs constitute a genetic determinant of pathogenesis for other retroviruses. However, this possibility has never been tested for BLV. Analyses to test correlation between clinical and genotypic traits across species must be corrected by including the group phylogeny. Otherwise, shared evolutionary history can jeopardize statistical independence. Thus, the influence of BLV LTR genetic variation on the clinical manifestation of the disease was investigated by employing Cladistic and Probabilistic, phylogenetic comparative methods.

With this purpose, the 5' LTR region of 40 BLV proviruses from bovines with different clinical presentations (AL, PL, LS) was sequenced. Seven polymorphic positions showing an apparent association with the clinical presentation were identified. A provirus phylogeny was obtained using env gene sequences from 28 of the 40 provirus studied in this work. Both Cladistic and Probabilistic comparative analyses based on the empirical sequence alignment and the provirus phylogeny suggested that positions 41 and 56 might be correlated to the clinical presentation. The probabilistic analysis further indicated an association with the viral pathogenesis for positions 373, 450, 494 and 505, though the corresponding statistical supports were lower in comparison to the supports obtained for positions 41 and 56. These observations indicate that the BLV LTRs might contain pathogenicity determinants.

